# Vitamin D Binding Protein Impact on 25-Hydroxyvitamin D Levels under Different Physiologic and Pathologic Conditions

**DOI:** 10.1155/2014/981581

**Published:** 2014-04-28

**Authors:** Pegah Yousefzadeh, Sue A. Shapses, Xiangbing Wang

**Affiliations:** ^1^Division of Endocrinology, Metabolism & Nutrition, Department of Medicine, Rutgers University-Robert Wood Johnson Medical School, New Brunswick, NJ 08903, USA; ^2^Department of Nutritional Sciences, Rutgers University, New Brunswick, NJ 08901, USA

## Abstract

There is a high prevalence of vitamin D deficiency worldwide, but how to define vitamin D deficiency is controversial. Currently, the plasma concentration of total 25-hydroxyvitamin D [25(OH)D] is considered an indicator of vitamin D status. The free hormone hypothesis states that protein-bound hormones are inactive while unbound hormones are free to exert biological activity. The majority of circulating 25(OH)D and 1,25(OH)_2_D is tightly bound to vitamin D binding protein (DBP), 10–15% is bound to albumin, and less than 1% of circulating vitamin D exists in an unbound form. While DBP is relatively stable in most healthy populations, a recent study showed that there are gene polymorphisms associated with race and ethnicity that could alter DBP levels and binding affinity. Furthermore, in some clinical situations, total vitamin D levels are altered and knowing whether DBP is also altered may have treatment implications. The aim of this review is to assess DBP concentration in different physiological and pathophysiological conditions. We suggest that DBP should be considered in the interpretation of 25(OH)D levels.

## 1. Introduction

Reports of the worldwide prevalence of low vitamin D status vary depending on the level of total 25-hydroxyvitamin D [25(OH)D] that is used to define vitamin D sufficiency. The plasma concentration of total 25(OH)D is considered an indicator of vitamin D status due to its long half-life of 15–35 days and lack of hormonal control of the hepatic 25-hydroxylase [1]. The free hormone hypothesis states that protein-bound hormones are relatively inactive while hormones not bound to binding proteins are available to exert biological activity [2]. The majority (85–90%) of circulating 25(OH)D and 1,25(OH)_2_D is tightly bound to DBP, with a smaller amount (10–15%) bound to albumin. Less than 1% of circulating vitamin D exists in a free, unbound form [3, 4]. It is difficult to define vitamin D deficiency because circulating 25(OH)D levels vary in different physiologic and disease states. It is unclear whether a change in levels of DBP would have any effect on concentrations of vitamin D metabolites, as DBP circulates at a higher level than its ligands. In a recent study, it was reported that despite the lower levels of total 25(OH)D and DBP in blacks compared to whites, both groups had similar concentrations of estimated bioavailable 25(OH)D and can be explained by a difference in polymorphisms in the DBP gene [5]. Another recent study used a novel immunoassay to directly measure free 25(OH)D, and they concluded that the current algorithms to calculate free 25(OH)D may not be accurate and further investigation is required [6]. A new assay that measures free serum 25(OH)D directly is now available by the Future Diagnostics (Wijchen, The Netherlands), but it is labor intensive, costly, and time-consuming. Alternatively, the bioavailable and DBP-bound 25(OH)D can be calculated using equations adapted from Vermeulen et al. [7] and is currently being used in clinical practice [8]. Recent studies have found that serum DBP and vitamin D levels are decreased in type 1 diabetes mellitus (T1DM) [9], chronic liver [10], and renal diseases [11], while pregnancy and oral contraceptive pills (OCP) increase DBP and vitamin D levels [12, 13]. On the other hand, in a large cohort of healthy adults with sufficient vitamin D levels, the biological effects of vitamin D on PTH levels are mainly independent of DBP concentration [14]. This finding may provide useful information for studies investigating the relationship between 25(OH)D and DBP in different age groups, races, and disease states. Dynamic changes of DBP levels should be integrated into interpreting the serum levels of 25(OH)D in these conditions. Besides binding to vitamin D metabolites, DBP has other physiological functions, including interacting with target organ or cells. In addition, megalin, possibly in conjunction with cubilin, mediates uptake of DBP-bound 25(OH)D into the kidney for the intracrine conversion of 25(OH)D to 1,25(OH)D [4]. Megalin may also play a role in extrarenal tissues [4]. The aim of this review is to discuss the correlation between serum levels of DBP and 25(OH)D in various physiologic and pathologic conditions.

## 2. Physiological Factors Affecting DBP and 25(OH)D Levels

### 2.1. Race and DBP Genotype

There is a higher prevalence of vitamin D deficiency among African Americans, as their total 25(OH)D levels are lower than those in white individuals. This could be due to their lower cutaneous synthesis of vitamin D [15]. Levels of total 25(OH)D are in part genetically determined. Variations in the DBP originally referred to as GC1F, GC1S, and GC2 were first reported more than 50 years ago and may be associated with changes in binding affinity or serum concentration of DBP. The protein variants are now recognized as resulting from polymorphisms in the DBP binding protein gene GC. The phenotypic variations in the DBP amino acid sequence are distinguished by single nucleotide polymorphisms (SNPs) rs7041 and rs4588. Blacks and Asians are more likely to carry GC1F DBP, which has the highest affinity for 25(OH)D and is associated with low DBP levels. Whites are more likely to carry GC1S DBP. GC2, which has a lower affinity for 25(OH)D and is associated with higher DBP levels, is frequently found in whites and rarely found in blacks [4, 5]. The high prevalence of GC1F in blacks results in concentrations of bioavailable 25(OH)D similar to those in whites [5]. Engelman et al. [16] showed that homozygosity for the CG1F allele (DDTT) occurred in 53% of African Americans but only 6% of Caucasians and 13% of Hispanics. The rs4588 and rs7041 SNPs were associated with 25(OH)D levels in Hispanics and African Americans. Combinations of these alleles appear to alter DBP concentration, the affinity for 25(OH)D, and 25(OH) levels [17, 18]. In a cross-section study, Santos et al. found that the AA genotype of rs4588 and TT genotypic of rs7044 and CT-AT/AT-AT (GC1f-2/2-2) were associated with lower 25(OH)D levels in healthy girl [19]. The rs4588 and rs7041 SNPs were associated with 25(OH)D levels and rs4588 was associated with 1,25(OH)_2_D levels in Hispanics and African Americans [16]. Cheung et al. [20] in a study of Southern Chinese women found that among four SNPs (rs2282679, rs10741657, rs12785878, and rs6013897), rs2282679 was associated with serum 25(OH)D levels and vitamin D insufficiency while rs12785878 was associated with vitamin D insufficiency only.

### 2.2. Age and Gender

Age appears to have an impact on DBP concentration, as it does on other globulins such as sex hormone binding protein (SHBG) and insulin-like growth factor binding protein (IGFBP). Vitamin D insufficiency is common among older adults. Our recent study showed that DBP was negatively correlated with age in female subjects indicating that age might be an independent factor affecting DBP and 25(OH)D levels [21]. Carpenter et al. [22] suggested that in young children genetic variance of the common rs4588 affects circulating levels of the DBP, which in turn affects circulating 25(OH)D levels in infants and toddlers. The GC genotype may be related to the susceptibility to low 25(OH)D levels in female children and adolescents. The GC genotype also affects circulating 25(OH)D independent of its effect on circulating DBP. Genome-wide association studies have found an association between variants in the GC genotypes and serum levels of 25(OH)D and PHT in older adults [23]. Winters et al. [24] found that DBP was not an important determinant of circulating 25(OH)D in women, nor was it affected by race or adiposity. In a study in which DBP levels were assessed in 100 healthy, middle-aged, and older participants, it was found that women had higher mean DBP levels than men, but no associations were observed between DBP levels and age, body weight, BMI, fat mass, or fat percentage [25]. Blanton et al. [9] confirmed that DBP levels were lower in males than in females, which could be due to estrogen effects on DBP.

### 2.3. Pregnancy and Estrogen

The increase in DBP levels associated with pregnancy results from the stimulation of DBP synthesis by estrogen [12]. It has been shown that while DBP is higher in pregnancy, free 25(OH)D is not lower, which can be attributed to weaker binding to DBP [26]. The use of OCP may also affect 25(OH)D concentrations and DBP levels. The limited data available on the effects of OCP on 25(OH)D concentrations suggest no change or an increase in total 25(OH)D [13], whereas most studies consistently report an increase in levels of 1,25(OH)_2_D and DBP [27].

DBP may be regulated by various hormone levels, as is the case with other globulins such as sex hormone binding globulin (SHBG) and thyroid binding globulin (TBG). Egawa et al. [28] reported that the DBP production might be regulated by insulin, estradiol, triamcinolone, dihydrotestosterone, or epidermal growth factor. The use of OCP is associated with 13%–25% higher concentrations of total 25(OH)D and 1,25(OH)_2_D and DBP. The estrogen component of OCP may increase DBP synthesis or decrease its catabolism. The authors suggest that use of OCP should be considered in the interpretation of plasma concentrations of vitamin D metabolites [13]. Our recent preliminary findings showed that DBP and 25(OH)D were lower in postmenopausal women compared with premenopausal women [21]. Consistent with this finding, the initiation of estrogen therapy in postmenopausal women caused a significant 8% increase in DBP levels [29] also suggesting that estrogen is a factor affecting DBP and 25(OH)D levels.

### 2.4. Obesity

The association between reduced 25(OH)D concentrations and obesity is well established, although the mechanisms for the lower 25(OH)D concentrations are not fully described. In a recent report, it was shown that DBP was lower in obese adolescents compared to those with normal weight and there was a positive correlation between DBP and 25(OH)D levels. The authors also reported that there was an inverse relationship between insulin levels and DBP levels in obese adolescents even when corrected for adiposity. It was suggested that insulin suppresses the production of DBP [30]. Obese women had higher DBP concentrations and lower free 25(OH)D compared with normal-weight women and a lower free 25(OH)D level. The obese women were more likely to have 25(OH)D concentrations that could be considered suboptimal [31].

## 3. Pathologic Factors Affecting DBP and 25(OH)D Levels

### 3.1. Liver Disease

The liver is where 25-hydroxylation of vitamin D occurs and the majority of DBP is synthesized. The prevalence of vitamin D insufficiency is particularly high in patients with chronic liver disease. Vitamin D deficiency or low total vitamin D levels in chronic liver disease is likely to result from a number of mechanisms including lower levels of sun exposure and inadequate dietary intake of vitamin D. Serum 25(OH)D levels are inversely related to the severity of chronic liver disease [32]. Decreased production of DBP and albumin, which also carries vitamin D, might be critical in chronic liver disease [10, 33]. However, patients with cirrhosis and low albumin concentrations have higher free 25(OH)D levels [26]. Patients with end-stage liver diseases had a high prevalence of low total 25(OH) but maintained normal serum corrected calcium levels and did not develop secondary hyperparathyroidism [33]. Low total Vitamin D and low DBP levels in liver failure can be corrected with a liver transplant after which serum DBP and albumin increase substantially. Low DBP levels likely have a role in low total vitamin D levels in profound liver dysfunction [34].

### 3.2. Renal Disease

Low total vitamin D levels are common in patients with chronic kidney disease (CKD) at all stages. An alteration in megalin-dependent uptake of DBP in the kidney [4] in CKD would be expected to decrease the intracrine conversion of 25(OH)D to 1,25(OH)D leading to complications, such as renal osteodystrophy. Increased urinary excretion of DBP has been associated with tubular dysfunction, such as in patients with nephrotic syndrome [35] and renal Fanconi syndrome [36]. Increased urinary loss of DBP has been postulated to contribute to the low total vitamin D levels in patients with proteinuria. However, a recent study by Doorenbos et al. [37] found that antiproteinuric treatment reduced urinary loss of DBP, but did not affect 25(OH)D levels in CKD. This is consistent with the hypothesis that urinary DBP is a marker of renal interstitial inflammation and fibrosis [38]. When compared to white patients, black patients receiving hemodialysis had lower levels of total 25(OH)D, but similar levels of bioavailable 25(OH)D levels. Bioavailable 25(OH)D levels but not total 25(OH)D and 1,25(OH)_2_D were significantly correlated with serum calcium [39]. It has also been shown that children with CKD exhibit altered concentration of DBP and bioavailable 25(OH), and there is an important impact of their underlying disease [11]. These findings suggest that bioavailable vitamin D levels are better correlated with measures of mineral metabolism than total vitamin D levels in patients receiving hemodialysis.

### 3.3. Diabetes

Blanton et al. [9] showed that serum DBP levels are decreased in patients with T1DM compared to healthy controls. They suggested that multiple components in the metabolic pathway of vitamin D may be altered in T1DM and, collectively, have the potential to influence disease pathogenesis. Another study showed that urinary loss of DBP in T1DM was higher compared to healthy individuals suggesting that urinary loss of DBP might contribute to the lower levels of serum 25(OH)D [40]. Tian et al. [41] found that DBP was higher in diabetic patients with nephropathy and microalbuminuria or macroalbuminuria compared to healthy controls or diabetic patients with normoalbuminuria. They suggested that urinary DBP is a potential biomarker for early detection of diabetic nephropathy.

### 3.4. Primary Hyperparathyroidism (PHPT)

Low total 25(OH)D levels are commonly encountered in patients with PHPT. The causes are not totally understood, but proposed mechanisms include stimulation by PTH of the conversion of 25(OH)D to 1,25(OH)_2_D by 1*α*-hydroxylase and the increased catabolism of 25(OH)D by CYP24A1 induced by the high levels of 1,25(OH)_2_D that prevail in PHPT [42]. We previously reported that both serum 25(OH)D and DBP levels were significantly lower in female patients with PHPT compared with control subjects. DBP levels were inversely correlated with intact PTH, suggesting that PTH regulates DBP production [43]. The findings suggest that a low DBP level contributes to the low 25(OH)D level observed in female PHPT patients.

### 3.5. Cancer

There are several reports that there is an association between vitamin D deficiency and risk of developing cancer. A recent meta-analysis suggested an inverse association of 25(OH)D with total cancer incidence and mortality [44]. However, Weinstein et al. [45] reported that 25(OH)D levels were positively correlated with pancreatic cancer risk and DBP was inversely associated with pancreatic cancer risk. Higher DBP concentrations may result in increased bound 25(OH)D and reduced free 25(OH)D. These authors suggest that simultaneous examination of DBP and 25(OH)D may be important in determining the association of vitamin D and cancer risk. In another study, the same authors found that serum DBP was not associated with prostate cancer risk overall; however, high serum DBP was associated with significantly decreased risk of prostate cancer in men with lower 25(OH)D concentrations and increased risk in men with higher 25(OH)D. These data suggest that the primary vitamin D carrier protein DBP modulates the impact of vitamin D status on prostate cancer [46]. Together, these data suggest that the combination of both DBP and 25(OH)D is needed to understand cancer risk [45, 46]. Mondul et al. [47] reported that low serum 25(OH)D levels were associated with a higher risk of bladder cancer and provided data to support an etiologic role for vitamin D in bladder cancer. They suggested that free circulating vitamin D might be a more relevant factor when examining bladder and other cancers. Overall, the data for cancer is still emerging and the role of DBP requires further study.

### 3.6. HIV and Inflammation

Comparing over 200 HIV-infected youth on stable treatment with regimens containing tenofovir disoproxil fumarate (TDF) to those whose treatment lacked TDF, Havens et al. [48] found that the higher plasma tenofovir concentrations were associated with higher DBP and lower free 1, 25(OH)_2_D. This might imply a functional vitamin D deficiency and can explain the TDF-associated increase in parathyroid hormone.

It is also common to see vitamin D insufficiency in hospitalized patients with an acute illness. DBP level is decreased in acute inflammatory conditions. One study showed that, following surgery, the mean CRP increased but both serums 25(OH)D and DBP decreased [49]. Patients with sepsis have a high mortality rate as well as a high prevalence of vitamin D deficiency. In addition, septic patients have decreased DBP levels, which further exacerbate the total low vitamin D levels but might attenuate the risk of vitamin D deficiency by maintaining normal free or bioavailable 25(OH)D [50]. Jeng et al. found that 25(OH)D and DBP levels were lower in patients in the intensive care unit with sepsis compared with healthy controls [51]. Serum 25(OH)D may be an unreliable biomarker of vitamin D status after acute inflammatory insult.

### 3.7. Procedures

Plasma exchange is a therapeutic procedure that is used to remove pathogenic circulating proteins from patients' plasma, and it often results in the depletion of physiologically important proteins including DBP. Plasma exchange induced an acute reversible decrease in plasma 1, 25(OH)_2_D, DBP, and calcium and a sustained decrease in plasma 25(OH)D [52]. Another study showed that, in CKD stages 4-5 children on peritoneal dialysis (PD), urinary DBP losses were >300-fold higher than seen in age-matched healthy children and correlated with urinary albumin loss. They concluded that peritoneal DBP losses mirror both dialysate and urinary albumin losses and are associated with a longer dialysis vintage but do not contribute to vitamin D deficiency in children on PD [53]. These findings suggest that serum 25(OH)D may be an unreliable biomarker of vitamin D status after procedures such as plasma exchange and PD.

## 4. Conclusion

Different physiologic and pathologic conditions can affect DBP levels, which in turn affect circulating 25(OH)D levels. Therefore, alterations in DBP levels should be considered as potential confounders on the interpretation of plasma total 25(OH)D concentrations. Further research is required to assess whether the free 25(OH)D index as compared to total 25(OH)D levels is a better marker of 25(OH)D tissue availability and if it has a higher correlation with indices of skeletal and nonskeletal outcomes.
